# Sensitization to common allergens among patients with allergies in major Iranian cities: a systematic review and meta-analysis

**DOI:** 10.4178/epih.e2017007

**Published:** 2017-02-05

**Authors:** Mozhgan Moghtaderi, Saeed Hosseini Teshnizi, Shirin Farjadian

**Affiliations:** 1 Allergy Research Center, Shiraz University of Medical Sciences, Shiraz, Iran; 2 Allergy Clinic of Ali-Asghar Hospital, Shiraz University of Medical Sciences, Shiraz, Iran; 3 Clinical Research Development Center of Children Hospital, Hormozgan University of Medical Sciences, Bandar Abbas, Iran; 4 Department of Immunology, Shiraz University of Medical Sciences, Shiraz, Iran

**Keywords:** Allergens, Cockroaches, Fungi, Pollen, Hypersensitivity, Iran

## Abstract

Various allergens are implicated in the pathogenesis of allergic diseases in different regions. This study attempted to identify the most common allergens among patients with allergies based on the results of skin prick tests in different parts of Iran. Relevant studies conducted from 2000 to 2016 were identified from the MEDLINE database. Six common groups of allergen types, including animal, cockroach, food, fungus, house dust mite, and pollen were considered. Subgroup analysis was performed to determine the prevalence of each type of allergen. The Egger test was used to assess publication bias. We included 44 studies in this meta-analysis. The overall prevalence of positive skin test results for at least one allergen was estimated to be 59% in patients with allergies in various parts of Iran. The number of patients was 11,646 (56% male and 44% female), with a mean age of 17.46±11.12 years. The most common allergen sources were pollen (47.0%), mites (35.2%), and food (15.3%). The prevalence of sensitization to food and cockroach allergens among children was greater than among adults. Pollen is the most common allergen sensitization in cities of Iran with a warm and dry climate; however, sensitization to house dust mites is predominant in northern and southern coastal areas of Iran.

## INTRODUCTION

The term ‘allergen’ is used to describe any substance capable of stimulating production of immunoglobulin E (IgE) in a genetically predisposed individual [1]. The first allergens were identified in the 1980s. Thus far, many allergens have been reported by cross-referencing databases, such as the Allergome and Pfam databases [2,3]. The most clinically important allergens are those related to animals, cockroaches, house dust mites (HDMs), foods, fungi, pollens, latex, and venom. Outdoors allergen sources are likely to be pollens, while HDMs are the most common source of indoor allergens [4].

Most animal allergens belong to mammals and birds, which are easily spread into the environment [5-7]. Sensitization to cockroaches is among the most common factors contributing to increased asthma morbidity. It appears that genetic background may also play a role in susceptibility to cockroach allergens [8,9]. HDMs are an important factor that may exacerbate different types of allergic diseases in predisposed individuals. These small arthropods live in close contact with humans and are found in large numbers in beds, sofas, carpets, and furniture. The growth of mites is inhibited in environments with low humidity and extreme temperatures [10,11]. Food-allergic patients, mainly children, show a higher sensitization to the ingestion of eggs, cow’s milk, nuts, soy, seafood, and wheat. However, allergies to various fruits and vegetables have also been reported, with lower frequencies [12,13]. Fungi are considered to be a primarily outdoor source of allergens, but the *Aspergillus* species can be found inside in warm and humid places [14,15]. Pollen allergens arise from the pollination processes of trees, weeds, and grass species. In many regions around the world, trees compromise the most clinically relevant source of allergenic pollens. Weeds can be defined as unwanted plants, and grasses are ubiquitous plants, which account for over 95% of allergies [16-18].

As a diagnostic test, the skin prick test (SPT) is routinely used to detect IgE-mediated sensitization to specific allergens. The SPT procedure is performed using standard commercial extracts of allergens administered with a sterile lancet on the forearm. The results of the skin test are examined after 15 minutes and considered positive when the wheal is 3 mm greater in diameter than the negative control (saline) [19].

Iran is the second largest country in the Middle East and 18th largest in the world, with a total area of 1,648,195 km^2^ and a population of around 78.4 million in 2016. Iran has a hot and dry climate in most areas, but there is high humidity on the northern coastal area along the Caspian Sea, and the southern coastal area adjacent along the Persian Gulf. Iran is divided into 31 provinces. Tehran (including Karaj), Mashhad, Isfahan, Tabriz, Shiraz, and Ahvaz are the five largest cities.

This systematic review and meta-analysis was designed to determine the prevalence of sensitization to common allergens using the published data on allergen sensitization in different regions of Iran.

## MATERIALS AND METHODS

### Search method

The literature and reference searches were performed in March 2016. The present study was designed based on the guidelines for the Preferred Reporting Items for Systematic Reviews and Meta-Analyses (PRISMA) statement [20]. Persian-language and English-language databases were accessed and searched for articles that were published in the period from January 2000 to March 2016 published from January 2000 to March 2016. Articles from the MEDLINE database, as well as Iranian databases (Magiran, Iran Medex) and international databases (PubMed, ProQuest, Scopus SID, Google Scholar, and Science Direct) were identified.

The databases were searched based on the following keywords: (1) [“allergens” AND “Iran”], (2) [“asthma” AND “Iran”], (3) [“allergic rhinitis” AND “Iran”], (4) [“atopic dermatitis” AND “Iran”] and [“skin test” AND “Iran”]. The reference sections of all included studies were also utilized to identify additional relevant articles.

### Data extraction

To assess studies for inclusion in the meta-analysis, two authors (MM and SHT) separately screened the title and abstract and then reviewed the full texts of studies identified in the literature review. A 93% match in the assessment of article inclusion was obtained between the two authors, which showed that there was a high agreement between authors. Any disagreements were resolved by consultation with a third author (SF) for the final decision.

To assess the quality of studies included in the meta-analysis, we selected six items from the Strengthening of the Reporting of Observational Studies in Epidemiology (STROBE) checklist and assessed studies for whether they: (1) clearly defined the outcome (i.e., allergen), (2) gave the eligibility criteria, (3) presented key elements of study design, (4) reported numbers of outcome events, (5) explained how the study sample was determined, and (6) described the setting, locations, and relevant dates of the study. The studies that fulfilled all criteria were classified as high quality. The studies that did not fulfill one of the criteria were classified as intermediate quality, and the studies that did not fulfill more than one of the criteria were classified as low quality.

We extracted the following information: the name of the first author, the publication year of the article, the province in which the study was performed, the type of study, the sample size of the study, and the age and sex of the patients. The rate of sensitization to at least one of the allergens, including those of animals, cockroaches, food, fungi, HDMs, and pollen, was also reported.

### Inclusion and exclusion criteria

Studies were included in this meta-analysis if the following criteria were met: (1) the study was conducted between January 2000 and March 2016; (2) it provided sufficient information to estimate the prevalence of allergens, with standard errors (SEs) and confidence intervals (CIs); (3) it reported descriptive statistics of age and sex and the prevalence of at least one of the allergens out of those of animals, cockroaches, food, fungi, HDMs, and pollen in terms of types of characteristics; (4) it was a cross-sectional study; (5) it was published in Persian or English; and (6) the study participants had a primary diagnosis for an allergy, including asthma, allergic rhinitis, atopic dermatitis, food allergy, chronic sinusitis, or chronic urticaria.

Studies were excluded if: (1) the study was a randomized controlled trial, a case report, or an animal study; (2) the article did not offer sufficient data to calculate the estimated prevalence of the allergen; (3) the study reported venom or latex allergens (these studies are addressed in the discussion); or (4) the article overlapped with another that had been identified.

### Statistical analysis

We present the effect size for each study as prevalence (P). The SE of each study was calculated based on a binomial distribution. The results for each study and the pooled outcomes were displayed as forest plots (reported as an effect estimate with the 95% CI). To assess heterogeneity among studies, the I-square statistic (I^2^) (with 25% designated as low, 50% as medium, and 75% as high heterogeneity) as well as the results of the Cochran Q-test (with p< 0.1 considered to be statistically significant) were also reported. Subgroup meta-analysis was used to compare the prevalence of allergens based on the results of SPTs among different age groups. The Egger test was used to evaluate the publication bias, plotting the regression line between the precision of the studies (the independent variable) and the standardized effect (the dependent variable). Stata version 11 (StataCorp., College Station, TX, USA) was used for data analysis.

## RESULTS

### Literature search

**Figure 1.**
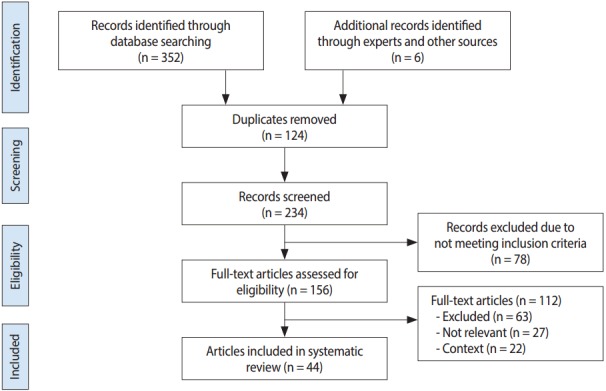
Flow diagram for study inclusion in the systematic review and meta-analysis.

Initial searches of databases identified 352 articles and an additional six studies through hand searches and expert suggestions, giving a total of 358 articles that were screened. Out of these, 156 were chosen for reading of the full text and 44 were included in this meta-analysis. Figure 1 shows our article selection process according to the PRISMA flowchart.

### Characteristics of the studies

All studies reported in this systematic review and meta-analysis were cross-sectional studies. After all inclusion and exclusion criteria had been evaluated, 44 studies containing 11,646 patients (56% male and 44% female) were included in the meta-analysis [21-64]. The mean age of the patients was 17.7 years (95% CI, 14.0 to 21.4 years). Of the 44 selected research studies, animal allergens were examined in 15, cockroach allergens in 17, food allergens in 21, fungal allergens in 23, HDM allergens in 25, and pollen allergens in 22. The basic characteristics of the published studies are presented in Table 1.

**Figure 2.**
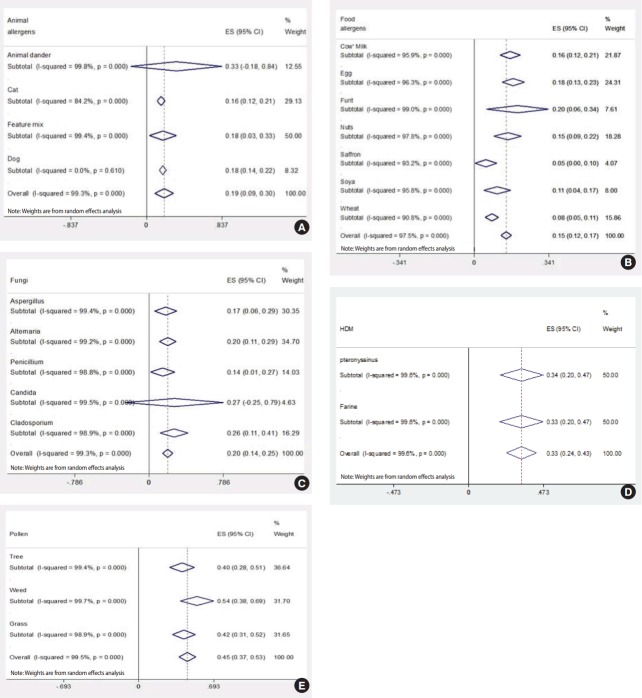
Forest plot of the prevalence of (A) animal, (B) food, (C) fungi, (D) house dust mite (HDM), and (E) pollen allergens sensitization among Iranian patients with allergies. ES, effect size; CI, confidence interval.

The prevalence of various animal allergens among patients with allergies is shown in Figure 2A. These patients most often showed sensitization to animal dander (33%). Other types of animal allergen showed a similar prevalence rate (about 18%).

The prevalence of sensitization to food allergens is shown in Figure 2B. A positive skin test result for cow’s milk, eggs, and nuts was obtained in 16, 18, and 15% of cases, respectively. In addition, overall sensitization to food allergens was 15%.

As shown in Figure 2C, the most common fungal allergen was *Candida* (27%), followed by *Cladosporium* (26%), *Alternaria* (20%), and *Aspergillus* (17%).

Sensitization to HDMs was reported in 33% of cases, as shown in Figure 2D. No significant differences were found between the prevalence of *Dermatophagoides pteronyssinus* and *Dermatophagoides farina* sensitization in patients with allergies.

As shown in Figure 2E, the overall sensitization to pollen allergens was 45% in patients with allergies. Sensitization to weed allergens had the highest prevalence (54%), followed by grass (42%), and trees (40%).

The estimated frequency of common allergens in major cities of Iran is shown in Table 2 according to the results of the subgroup analysis. The highest sensitization to animal allergens and HDMs was detected in coastal cities. The highest prevalence of cockroach allergen sensitization was reported in Shiraz and Ahvaz. Sensitization to different food allergens was approximately the same between major cities. The highest prevalence of fungal allergen sensitization was in Tehran. The frequency of sensitization to pollen was highest in Ahvaz, Mashhad, and Shiraz, in that order (Table 2).

The age distribution of patients with positive SPT results for common allergens is given in Table 3. Cockroach allergen sensitization was more prevalent in children ≤ 16 years old than in adults. The prevalence of sensitization to food allergens was also significantly higher in patients younger than 5 years old compared to those of older age, at 26.2% (95% CI, 22.8 to 29.7%). However, the prevalence of sensitization to other allergens such as animal, fungal, HDM, and pollen was more prevalent among adults (Table 3).

The results of the Egger test for each of subgroup of allergens showed that there was no publication bias among the studies (p> 0.05).

## DISCUSSION

An allergy is defined as an allergen-specific hypersensitivity disease. We estimated the prevalence of six common types of allergens, including those of animals, cockroaches, foods, fungi, HDMs, and pollens in patients with allergies in major cities of Iran. The estimated prevalence of sensitization to at least one of these allergens was found to be 59% among allergic patients, according to SPT results. However, because of the insufficiency of the data in some cities, such as Isfahan, we could not report the prevalence of sensitization to allergens in Iran in precise detail. Furthermore, not all patients in these selected studies were tested for all the considered allergens.

Our study demonstrated that the prevalence of a positive SPT result in allergy patients in Iran was generally consistent with results found in most neighboring countries. An analysis of SPT reactions in patients with allergic rhinitis yielded a positive result of 61% in Turkey [65], 69% in Pakistan [66], and 75% in Riyadh [67]. A large difference was observed in positive SPT results among Russian patients, at 21.8% [68].

Keeping a domesticated dog at home and direct exposure to this animal is very much limited in Iran because of religious beliefs; however, sensitization to animal allergens (cat, dog, and feather allergens in combination) was present in about 20% of cases among allergic patients. Direct contact with animals may lead to sensitization, but animal allergens can be transferred into environments that were never occupied by the animals [69]. Animal hypersensitivity was detected in 26% of Turkish patients with allergies, a country that shows a low pet ownership rate similar to Iran [70]. In the current study, the highest prevalence of sensitization to animal allergens was in coastal areas. This difference can be explained by the administration of SPTs with animal dander extract, including a broader type of animal allergens, in a study in Bushehr [35].

The overall prevalence of sensitization to cockroach allergens was 25% among Iranian patients with allergies. The prevalence of cockroach allergen sensitization was reported to be 12% in 337 tested children in Turkey [71], 33% in 151 asthmatic patients in Saudi Arabia [72], and 68.4% in asthmatic patients in Russia [73]. It seems that sensitization to cockroach allergens is more common among patients with asthma than in other cases of patients with allergies. Positive reactions to cockroach allergens were most frequent in allergic patients living in Ahvaz and Shiraz; this might be accounted for by a high level of cockroach infestation due to the warm climate in southern Iran. Children had more frequent positive results for sensitization to cockroaches; therefore, the application of insecticides and vigorous cleaning of children’s bedrooms may be needed to decrease sensitization among children.

Our included studies showed that, according to SPT results, the prevalence of sensitization to food allergens was 15% overall in patients with allergies. The prevalence of sensitization to food allergens was highest in Australia (83%) and the UK (74%). The reason for the lower incidence of food sensitization in Iranians may be the variation in their genetic background. A lower prevalence of food sensitization among adults compared to infants may be a result of a natural increase in tolerance with increasing age [74,75]. The most common sources of food allergens were eggs (18%) and cow’s milk (16%), followed by nuts (15%) and wheat (8%); this frequency is in accord with what has been found in other studies [76,77]. It is noteworthy that the specificity of the SPT is generally low for food allergens, partly because of enzymatic degradation of food proteins during the preparation of extracts, as well as cross-reactions between some food groups [19]. Saffron, a spice which is mostly cultivated in Iran, resulted in an immediate positive skin reaction in 70% of the patients in the study of Varasteh et al. [64]. No significant difference in the sensitization to food allergens was found among major cities of Iran.

The prevalence of sensitivity to various fungal allergens was reported to be from 5 to 20% in Iranian patients with allergies [78]. There are two reports on the prevalence of allergies to *Candida* in Iran: one reported a 0.7% prevalence in patients with rhinitis in Shiraz, and the other reported a 53% prevalence in patients with eczema and asthma in Tehran [41,44]. *Candida* is a commensal normal flora of the skin, the gastrointestinal tract, and the genitourinary system, although the significance of *Candida* in allergies is still controversial in the literature [79]; meanwhile, cross-reactivity to other fungal allergens should be considered [80]. Sensitization to other fungal allergens, including *Cladosporium, Alternaria, Aspergillus*, and *Penicillium*, was highest in Tehran and coastal areas because of the climate conditions.

The prevalence of sensitization to HDM allergens in Iranian patients with allergies was 33% in this study. The prevalence of positive results for sensitization to HDMs in neighboring countries has been reported as 25% in Turkey [81], 46% in the United Arab Emirates [82], and 67% in Russia [83]. The prevalence of sensitization to mites in patients with allergies living at sea level was higher than in those living in the high altitude areas of Iran, because mites grow better in environments of higher humidity [84].

In Iranian patients, the most common positive SPT result for allergen sensitization was for allergens from weed, grass, and tree pollen (45%) in all cities except coastal areas. Recent studies have shown the impact of climate change on aeroallergen acculturation, as global warming increases pollen production by plants and the allergenicity of pollens, lengthens the production period, broadens the distribution of pollens, and induces changes in plant biomass [85,86]. The results of subgroup analysis showed that the prevalence of sensitization to pollens was higher in southwestern Iran, including Ahvaz and Shiraz, than in other regions of Iran. Southwest Iran has a dry and hot climate with short winters, resulting in an increase in the production period and distribution of pollens. In recent years, the effects of storms in the Middle East have become a problematic phenomenon for allergic diseases in southern provinces of Iran [87].

There are other allergens from diverse sources associated with allergic disease. Latex products are occupationally associated aeroallergens, with the clinical significance of this allergen varying according to patient subgroups. The prevalence of sensitization to latex allergen in Iran was reported from 18 to 38% in health care personnel [88-90], and no sensitization was observed in workers in latex glove factories [91].

There has been no report of the prevalence of insect-sting allergies or their mortality in Iran. Bemanian et al. [92] suggested a relatively fast and safe protocol for venom immunotherapy in 10 patients with systemic reaction to honeybee or yellow jacket bee stings.

No significant publication bias was observed among published studies; both studies reporting low prevalence and high prevalence were included in this meta-analysis.

A positive skin test is not sufficient to confirm the presence of an allergic disease; however, it shows allergic sensitization, which necessitates the evaluation of clinical symptoms, and it also may predict the subsequent onset of allergic symptoms [86]. One of the sources of heterogeneity among allergens may have been differences in the age range of patients who were enrolled in the different studies included in this meta-analysis. The published data were insufficient for consideration of poly-sensitization to diverse allergens.

## CONCLUSION

This study estimated the reported prevalence of sensitization to at least one of the considered allergens to be 59% based on SPT results. In most parts of Iran, where the climate is hot and dry, pollens were the most common type of allergen, whereas sensitization to mites was most common in northern and southern coastal areas of Iran. This study will help in selecting panels of the most common allergens for SPTs and will also help in finding the best species of allergens for immunotherapy in this area.

## Figures and Tables

**Table 1.** Characteristics of studies included in the meta-analysis of the prevalence of sensitization to common allergens in Iran

Authors (publication year)	City	No. of patients	Age (mean)	Male (%)	Animal (%)	Cock-roach (%)	Food (%)	Fungal (%)	HDM (%)	Pollen (%)
Abdollahi-Fakhim et al. (2014) [21]	Tabriz	106	6.5	50	12	NR	26	10	23	35
Ahanchian et al. (2016) [22]	Mashhad	371	5.3	54	NR	NR	100	NR	NR	NR
Ahmadiafshar et al. (2008) [23]	Zanjan	200	28.2	44	12	15	9	12	16	41
Akbari Hedayat et al. (2000) [24]	Isfahan	1,077	NR	NR	NR	20	NR	37	35	48
Mokhtari Amirmajdi et al. (2011) [25]	Mashhad	58	29.8	45	NR	NR	NR	53	NR	NR
Arshi et al. (2010) [26]	Tehran	245	26.4	48	NR	NR	NR	50	64	92
Assarehzadegan et al. (2013) [27]	Ahvaz	299	32.0	52	NR	30	NR	24	43	89
Behmanesh et al. (2010) [28]	Mashhad	133	7.8	62	9	6	NR	NR	19	57
Bemanian et al. (2012) [29]	Yazd	95	22.7	55	NR	13	NR	NR	8	NR
Bonyadi et al. (2014) [30]	Tabriz	90	29.0	44	NR	NR	NR	24	NR	NR
Farajzadeh et al. (2010) [31]	Kerman	51	<5.0	63	NR	NR	62	NR	NR	NR
Farhoudi et al. (2002) [32]	Tehran	100	6.2	68	2	29	10	NR	31	33
Farhoudi et al. (2005) [33]	Tehran	226	13.5	55	NR	25	NR	NR	19	62
Farjadian et al. (2012) [34]	Shiraz	79	3.0	56	NR	NR	15	NR	NR	NR
Farrokhi et al. (2015) [35]	Bushehr	743	27.2	53	78	NR	NR	82	88	77
Fazlollahi et al. (2007) [36]	Tehran	250	11.7	52	NR	NR	14	NR	NR	NR
Fereidouni et al. (2009) [37]	Mashhad	306	25.6	47	NR	21	NR	8	20	77
Fouladseresht et al. (2014) [38]	Kerman	157	NR	49	7	12	21	6	8	22
Ghaffari et al. (2012) [39]	Sari	375	13.5	34	7	15	NR	3	25	4
Hosseini et al. (2014) [40]	Tehran	313	5.7	62	15	18	21	26	22	26
Kashef et al. (2003) [41]	Shiraz	212	18.2	50	NR	NR	NR	8	22	92
Khazaei et al. (2003) [42]	Zahedan	1,285	2.0-79.0	43	70	NR	30	65	89	43
Khazaei et al. (2015) [43]	Zahedan	478	15.0-70.0	58	26	25	NR	53	51	30
Khosravi et al. (2009) [44]	Tehran	180	NR	NR	NR	NR	NR	54	NR	NR
Mahram et al. (2013) [45]	Qazvin	163	24.6	42	25	NR	NR	26	20	58
Moghtaderi et al. (2010) [46]	Shiraz	230	6.3	76	NR	NR	NR	10	NR	NR
Moghtaderi et al. (2012) [47]	Shiraz	90	1.6	53	NR	NR	40	NR	NR	NR
Moghtaderi et al. (2015) [48]	Shiraz	50	32.0	20	NR	NR	58	NR	NR	NR
Moghtaderi et al. (2015) [49]	Shiraz	656	27.6	44	16	30	NR	16	34	64
Moghtaderi et al. (2015) [50]	Shiraz	200	21.1	35	36	NR	NR	NR	NR	NR
Mohammadi et al. (2008) [51]	Tehran	206	18.0	52	NR	NR	5	NR	NR	NR
Mohammadzadeh et al. (2012) [52]	Babol	180	6.8	52	NR	NR	NR	NR	61	NR
Movahedi et al. (2000) [53]	Tehran	400	19.0	52	NR	NR	NR	NR	NR	57
Nabavi et al. (2010) [54]	Semnan	298	10.0	NR	NR	NR	30	NR	NR	NR
Nabavi et al. (2010) [55]	Semnan	220	<18.0	60	NR	NR	NR	35	NR	NR
Nabavizadeh et al. (2013) [56]	Yasuj	184	23.7	65	23	35	33	48	8	37
Onsori et al. (2016) [57]	Tehran	282	25.0	40	NR	NR	31	NR	NR	NR
Pourpak et al. (2003) [58]	Tehran	190	4.8	58	NR	NR	53	NR	NR	NR
Pourpak et al. (2004) [59]	Tehran	119	0.1-12.0	NR	NR	NR	44	NR	NR	NR
Safari et al. (2009) [60]	Shiraz	92	2.7	64	18	25	19	10	27	27
Salehi et al. (2009) [61]	Tehran	100	2.7	71	NR	NR	39	NR	NR	NR
Shakurnia et al. (2014) [62]	Ahvaz	407	31.5	52	NR	32	NR	NR	23	NR
Shakurnia et al. (2013) [63]	Ahvaz	111	30.7	51	NR	38	NR	32	41	86
Varasteh et al. (2007) [64]	Mashhad	38	38.0	NR	NR	NR	70	NR	NR	NR

HDM, house dust mite; NR, not reported.

**Table 2.** Frequency of allergen sensitivity differentiated by type of allergen and major cities of Iran

Allergen types	Cites							All cities
	Tehran	Mashhad + Semnan	Tabriz + Zanjan + Qazvin	Shiraz	Ahvaz	Coastal areas^1^	Kerman + Yazd + Isfahan	
Cockroach	23.9	13.7	25.3	32.2	32.0	15.7	16.0	25.1
	(19.2, 28.6)	(3.7, 28.8)	(4.8, 45.8)	(24.4, 0.0)	(28.6, 35.5)	(7.7, 23.7)	(10.6, 21.4)	(20.1, 30.2)
Food	4.9	5.1	3.1	4.2	TFS	4.0	3.6	14.6
	(2.6, 7.2)	(3.2, 7.0)	(9.0, 7.2)	(4.0, 68.0)		(3.2, 4.8)	(0.8, 7.9)	(12.2, 17.0)
Fungi	40.0	3.2	9.4	10.3	13.1	33.5	TFS	19.6
	(13.5, 66.4)	(16.4, 52.2)	(6.0, 18.2)	(1.8, 18.7)	(3.0, 23.3)	(26.0, 39.8)		(14.2, 25.0)
Pollen	44.5	55.0	32.5	48.1	83.1	35.1	25.1	44.8
	(23.6, 65.3)	(7.8, 65.4)	(28.0, 37.0)	(22.2, 74.1)	(79.0, 86.7)	(4.8, 65.4)	(7.2, 52.0)	(37.1, 52.5)
Animal	13.7	9.0	14.5	15.5	TFS	48.1	5.4	19.5
	(8.9, 18.8)	(4.1, 13.9)	(10.7, 18.4)	(13.2, 17.9)		(18.0, 78.2)	(1.9, 8.9)	(9.1, 29.9)
HDM	34.1	17.5	16.1	25.3	40.8	63.2	16.7	33.4
	(13.4, 548)	(13.9, 21.0)	(9.8, 22.4)	(10.8, 39.8)	(26.3, 55.3)	(40.3, 86.1)	(7.3, 37.1)	(24.0, 43.0)

Values are presented as prevalence % (95% confidence interval). TFS, too few studies; HDM, house dust mite.

1 The coastal areas were defined as Babol, Bushehr, Sari, and Zahedan.

**Table 3.** The prevalence of allergens according to allergen type and age group

Allergens	No. of studies	Prevalence % (95% CI)			Q-test		
		<5 yr	5-16 yr	>16 yr	Value	I-square (%)	p-value
Animal	14	TFS	8.4 (4.6, 12.2)	35.8 (15.0, 56.7)	3.0	99.6	0.002
Cockroach	16	TFS	7.1 (6.1, 8.0)	3.2 (2.5, 3.9)	40.7	94.4	<0.001
Food	21	26.2 (22.8, 29.7)	11.3 (9.8, 12.9)	2.8 (2.1, 3.5)	265.8	95.8	<0.001
Fungi	15	TFS	4.2 (3.3, 5.1)	5.3 (4.6, 6.0)	3.3	78.3	0.07
HDM	23	TFS	5.2 (4.4, 6.0)	8.1 (7.4, 8.8)	4.3	93.2	<0.001
Pollen	21	TFS	3.7 (3.0, 4.4)	8.4 (7.7, 9.1)	85.7	98.3	<0.001

CI, confidence interval; TFS, too few studies; HDM, house dust mite.
